# Thyroidectomy Effects on the Body Mass Index and Thyroid-Stimulating Hormone: A Systematic Review and Meta-Analysis

**DOI:** 10.7759/cureus.54585

**Published:** 2024-02-20

**Authors:** Hyder Mirghani, Ahmad M Fnjan, Abdullah F Almalki, Ali F Almadan, Omar Abdullah M Alammar, Abdulaziz S Alhwiati, Amer A Laradhi, Ahmed M Bakour, Mohamad A Aljahed, Abdulmajeed M Alzahrani

**Affiliations:** 1 Department of Internal Medicine, University of Tabuk, Tabuk, SAU; 2 Department of Internal Medicine, King Abdulaziz University Hospital, Jeddah, SAU; 3 Department of Internal Medicine, King Fahad Hospital, Al Hofuf, SAU

**Keywords:** hyperthyroidism, thyroid malignancy, body mass index, weight, thyroidectomy

## Abstract

Thyroidectomy is common and is performed for malignancy, goiters with pressure symptoms, and certain types of Grave's disease. Weight and body mass index (BMI) following thyroidectomy were discussed controversially. This meta-analysis aimed to assess weight and BMI following thyroidectomy. A systematic literature search was conducted in PubMed, Medline, and Google Scholar with interest in articles that assessed body weight and BMI following total or subtotal thyroidectomy. The search engine was limited to the period from inception up to January 2024. Keywords "total thyroidectomy", "subtotal thyroidectomy", "Graves' disease", "multinodular goiter", "differentiated thyroid carcinoma", and "toxic nodules" were used. Out of the 634 articles retrieved, 89 full texts were screened, and only six studies (five retrospective and one prospective cohort) fulfilled the inclusion and exclusion criteria. No differences were evident regarding weight and BMI before and after thyroidectomy (odds ratio: -0.63, 95%CI: -1.50 to -0.24, P-value for the overall effect: 0.15; and odds ratio: -0.12, 95% CI: -0.41 to -0.16, P-value for the overall effect: 0.40 respectively). No heterogeneity was observed (*I*^2^ for heterogeneity: 0.0%). No association between thyroidectomy (when performed for differentiated thyroid carcinoma and hyperthyroidism), weight, and BMI was found. Further studies assessing thyroid-stimulating hormone (TSH) levels, radioactive iodine therapy, and thyroxine dose are needed.

## Introduction and background

Thyroidectomy (total or subtotal) is usually performed for malignancy, toxic multi-nodular goiter, Graves' disease, and goiter with pressure symptoms. In the United States of America, more than 75,000 thyroidectomies are performed annually [1,2]. Weight gain may be present even after reaching the targets of thyroid hormone replacement [3]. Weight gain is common among patients with hypothyroidism and is a major cause of poor quality of life and patient dissatisfaction [4,5]. Although thyroidectomy is safe and well-tolerated, specific consent forms are in practice to alert patients regarding thyroidectomy complications [6]. Post-thyroidectomy weight gain and asthenia were observed even among patients without hypothyroidism. Fatigue and increased body mass index (BMI) are major concerns for the patients and the treating physicians [7]. The knowledge of the effects of thyroidectomy on weight is vital for patients' shared decisions and informed consent [8]. Data on body weight and BMI after thyroidectomy are scarce. Therefore, this meta-analysis aimed to assess weight, BMI, and thyroid-stimulating hormone (TSH) changes following thyroidectomy.

Subjects and methods

Eligibility Criteria According to PICOS

This meta-analysis was conducted between December 2023 and January 2024 according to the Preferred Reporting Items for Systematic Reviews and Meta-Analyses (PRISMA) Guidelines.

Inclusion Criteria

All prospective and retrospective cohorts, case-control studies, and cross-sectional studies assessing body weight, BMI, and TSH changes following thyroidectomy were approached. Studies were eligible if they were published in English from the first published article up to January 2024.

Exclusion Criteria

Case reports, study protocols, editorials, letters to the editor, and animal studies, or otherwise published in a language other than English were not included. Studies that investigated body changes after thyroidectomy among euthyroid patients without the above-mentioned diseases were also excluded.

Outcome Measures

The primary outcomes were body weight and BMI following total or subtotal thyroidectomy and when indicated for differentiated thyroid carcinoma, Graves' disease, or multinodular goiter.

Information Sources and Search

Two researchers (H. M and I. A) undertook a systematic literature search; the search was conducted in PubMed, Medline, and Google Scholar with interest in articles that assessed body weight and BMI following total or subtotal thyroidectomy. In addition, the references list was searched for additional relevant studies. The search engine was limited to the period from inception up to January 2024. The keywords "total thyroidectomy", "subtotal thyroidectomy", "Graves' disease", "multinodular goiter", "differentiated thyroid carcinoma", and "toxic nodules" were used. Out of the 634 articles retrieved, 89 full texts were screened, and the Newcastle-Ottawa Scale was used to assess the risk of bias in the included studies [9]. However, only six studies [2,8,10-13] fulfilled the inclusion and exclusion criteria.

Data extraction

The data were entered in a structured table detailing the author's name, year of publication, country, patient's number, age, sex, type of study, duration of follow-up, and type and indication of thyroidectomy. The two authors cross-checked the collected data for any errors and discrepancies. Any discrepancies were solved by consensus. The Newcastle-Ottawa Scale was used to assess the risk of bias in the included studies [9].

Data analysis

The RevMan system for meta-analysis (version 5.4; Cochrane Collaboration, London, UK) was used, and the data were all continuous. Because no heterogeneity was observed, the fixed effect was used to compare BMI and weight before and after thyroidectomy. A P-value of <0.05 was considered significant.

**Figure 1:**
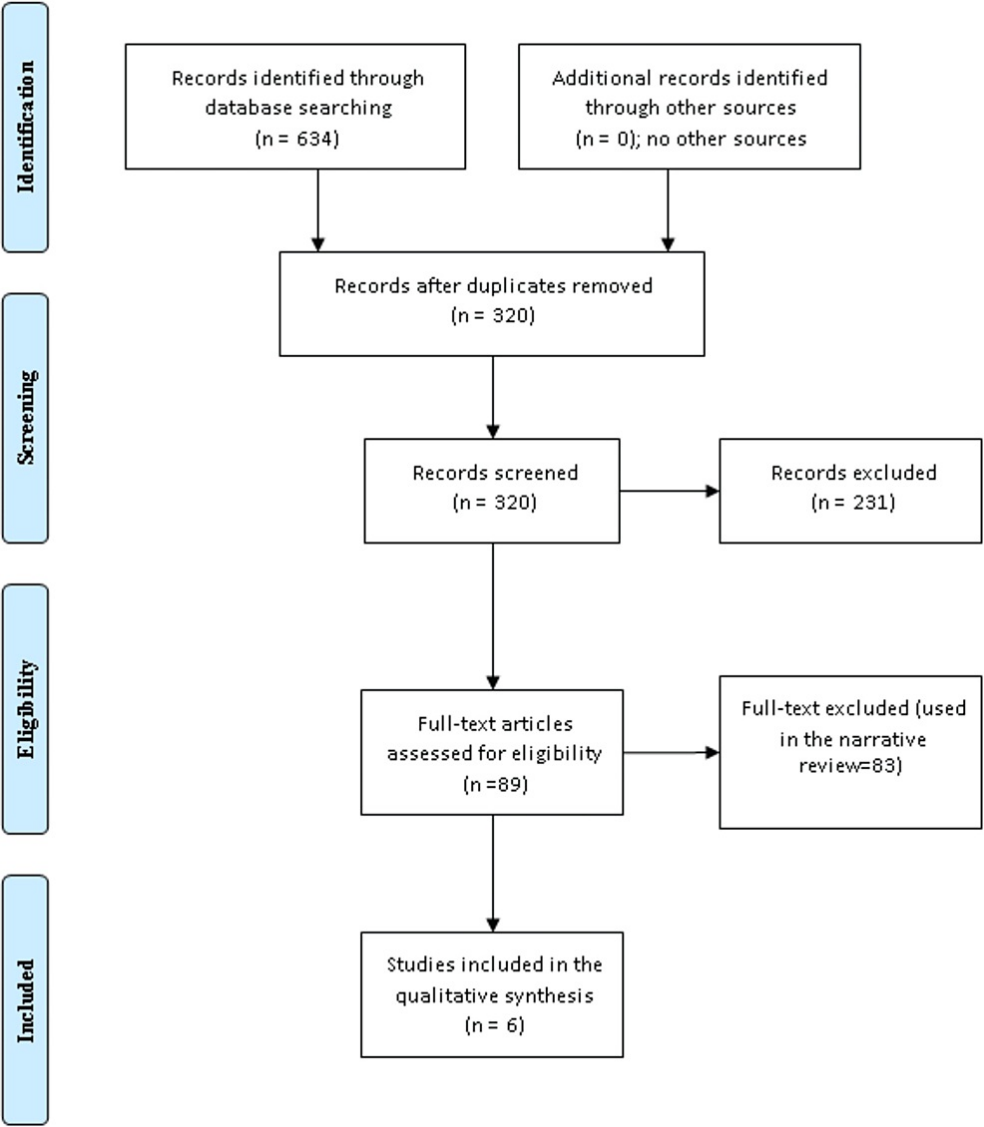
Weight, body mass index, and thyroid-stimulating hormone changes following thyroidectomy.

Figure 1 shows the systemic literature search according to the PRISMA guidelines. Out of the 634 articles retrieved, 320 articles remained after duplication removal, 89 full texts were screened, and only six studies fulfilled the inclusion and exclusion criteria.

## Review

Results

Figure 1 shows a PRISMA flow diagram summarizing the selection process for studies on weight and BMI change following thyroidectomy. This diagram outlines the systematic approach to study selection, ensuring a thorough and unbiased review of the literature. Table 1 presents the basic characteristics of the six included studies.

**Table 1:** Basic characteristics of the included studies.

Author	Country	Age/years (mean ± SD)	Sex	Type of Study	Diagnosis
Glick et al., 2014 [2]	Australia	55.8 ± 15.7	73.4% females	Retrospective, 107 patients	Multinodular goiter, Graves' disease, thyroid cancer and Hashimoto's thyroiditis
Ozdemir et al., 2010 [10]	Turkey	45.8	77.3% females	Prospective, 22 patients	Hyperthyroid
Park et al., 2019 [11]	Korea	48.2 ± 11.5	86.3% females	Retrospective, 227 patients	Cancer
Polotsky et al., 2012 [12]	USA	43	72% females	Retrospective, 153 patients	Cancer
Rotondi et al., 2014 [8]	Italy	Not mentioned	Not mentioned	Retrospective, 267 patients	Hyperthyroidism
Sohn et al., 2015 [13]	Korea	50.1 ± 10.3	71.4% females	Retrospective, 700 patients	Cancer

Table 2 illustrates the weight of patients and BMI before and after thyroidectomy and the duration of follow-up.

**Table 2:** Body weight and body mass index following thyroidectomy.

Author	Weight before in kg	Weight after in kg	BMI before (kg/m^2)	BMI after (kg/m^2)	Follow-up
Glick et al., 2014 [2]	78.8 ± 17.5	78.9 ± 17.6	N/A	N/A	18 months
Ozdemir et al., 2010 [10]	78.25 ± 12.9	79.75 ± 13.7	28.9 ± 4.1	29.45 ± 4.5	6 months
Park et al., 2019 [11]	62.4 ± 11.6	62.5 ± 11.7	24.4 ± 3.4	24.4 ± 3.4	28.3 months
Polotsky et al., 2012 [12]	76 ± 21	79 ± 23	26.9 ± 5.8	27.9 ± 6.60	60 months
Rotondi et al., 2014 [8]	70.8 ± 16.0	72.5 ± 16.4	N/A	N/A	9 months

Table 3 shows the Newcastle-Ottawa Scale of the included studies; all the studies were of good quality (>7).

**Table 3:** Newcastle-Ottawa Scale risk of bias of the included studies.

Author	Country	Selection bias	Comparability bias	Outcome	Total score
Glick et al., 2014 [2]	Australia	4	2	2	8
Ozdemir et al., 2010 [10]	Turkey	4	2	2	8
Park et al., 2019 [11]	Korea	4	2	2	8
Polotsky et al., 2012 [12]	USA	4	2	3	9
Rotondi et al., 2014 [8]	Italy	4	2	2	8
Sohn et al., 2015 [13]	Korea	4	2	2	8

In the present meta-analysis, six studies [2,8,10-13] comparing weight gain among patients with total or subtotal thyroidectomy were pooled using the fixed effect (1,472 patients were included). Two studies were from Asia, one from the USA, one from Europe, and one from Australia, and all were retrospective, except for Ozdemir et al. [10], which was prospective.

**Figure 2:**
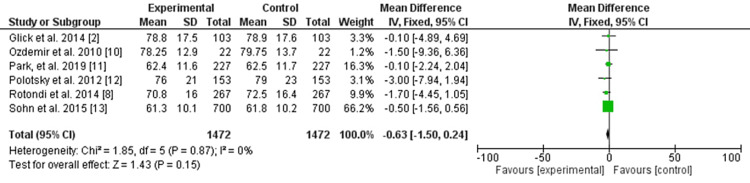
Weight change after thyroidectomy.

No differences between the two groups were evident regarding weight before and after thyroidectomy (odds ratio: -0.63, 95% CI: -1.50 to -0.24, significance tests for the weighted average effect size (Z): 1.43, and the P-value for the overall effect: 0.15). No heterogeneity was observed (P-value for heterogeneity: 0.87, chi-square: 1.85, standard difference: 5, and *I^2^* for heterogeneity: 0%) (Figure 2).

**Figure 3:**
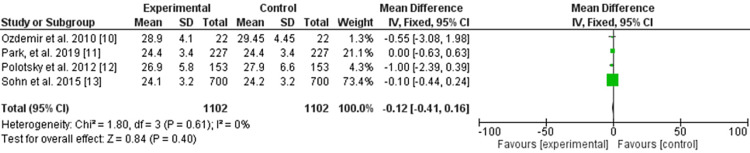
Body mass index change after thyroidectomy.

Regarding the BMI, four studies were included with 1,102 patients [10-13], and no difference was found after thyroidectomy (odds ratio: -0.12, 95% CI: -0.41 to -0.16, significance tests for the weighted average effect size (Z): 0.84, and P-value for the overall effect: 0.40). No heterogeneity was observed (P-value for heterogeneity: 0.61, chi-square: 1.80, standard difference: 3, and *I^2^* for heterogeneity: 0.0%) (Figure 3).

**Figure 4:**
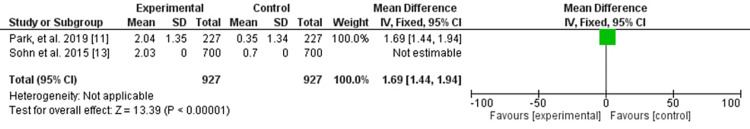
Thyroxine-releasing hormone levels pre- and post-thyroidectomy.

It is interesting to note that the results of two studies with 927 patients [11,13] showed a significant TSH reduction following thyroidectomy, test for overall effect (Z): 13.39, and P-value for the overall effect: <0001 (Figure 4).

Discussion

The underlying cause for thyroidectomy might affect weight change. Kyriacou et al. found more weight gain after severe hyperthyroidism, especially Graves' disease [14]. In the present meta-analysis, we included thyroid cancer in three studies [11-13] and hyperthyroidism in two studies [8,10], while one study included miscellaneous diagnoses [2]. The baseline thyroid hormone and young age were predictors of weight gain, rather than the extent of thyroid surgery [15,16]. In our previous meta-analysis, we found no effect of thyroidectomy on body weight and BMI. However, our previous study [17] was limited by the high heterogeneity observed and the fact that we included some studies using different thyroid treatments.

Few meta-analyses assessed weight changes following thyroidectomy with conflicting results [18,19]. In the current meta-analysis, no significant change in weight or BMI was observed. Our findings are similar to Singh et al. [19] in their review and meta-analysis who found that thyroidectomy did not contribute significantly to weight gain; the authors stated that patients with thyroid cancer and benign thyroid nodules gain weight of 1.07 kg and 1.5 kg, respectively [19]. The current findings were not in line with Huynh et al. [18] who found a significant weight gain following thyroidectomy, particularly among young patients with thyrotoxicosis [14]. Huynh et al.'s [18] study was limited by the significant heterogeneity of the included studies. A previous study found that post-menopausal women undergoing thyroidectomy gain more weight than their counterparts [20]. Post-operative dietary intervention might affect weight following surgery [14]. It is interesting to note that an early increase in weight (two to three months) highly predicts weight at six months post-operatively [8].

Many factors were suggested for weight and BMI changes following thyroidectomy including age, sex, T3 deficiency, TSH levels, and thyroid hormone withdrawal before radioactive iodine administration [2,15]. In the present meta-analysis, the majority of the patients were females of different ages (ranging from 43 to 55.8 years).

An interesting study followed thyroidectomized patients for three years and found no difference in weight between those with sub-clinical hyperthyroidism (induced by thyroxine replacement) and their counterparts who were euthyroid [16].

An interesting finding in a recent study published in Turkey found that 72.3% of patients gained weight after thyroidectomy, and the gain was mainly in fat mass and predicted by preoperative fat mass and high free T3 at baseline [21].

The high levels of TSH following thyroidectomy need special consideration because some differentiated thyroid cancers need thyroxine withdrawal for radioactive iodine. The high TSH might contribute to increasing fat mass [22]. In addition, high TSH is a risk factor for obesity and cardiometabolic disease.

Scerrino et al. [23] conducted a narrative review and suggested that the change in body weight following thyroidectomy might be explained by the change in patients' habits following thyroidectomy.

The strength of the current meta-analysis is that we included age, sex, and indication for surgery. In addition, we assessed the TSH levels before and after thyroidectomy. The small number of the included studies and including only observation studies limited the current study.

## Conclusions

No significant change was evident in body weight after thyroidectomy. In addition, the BMI following thyroidectomy for different thyroid diseases was not different. The thyroxine-releasing hormone was lower following thyroidectomy; the lower the TSH might be explained by thyroxine replacement. Physicians may need to educate their patients on this important belief for better decision-making.
